# Gut microbiome changes associated with chronic pancreatitis and pancreatic cancer: a systematic review and meta-analysis

**DOI:** 10.1097/JS9.0000000000001724

**Published:** 2024-06-07

**Authors:** Jiaze Hong, Yufan Fu, Xiaoqian Chen, Yurui Zhang, Xinyi Li, Tianhang Li, Yilin Liu, Mengke Fan, Rong Lin

**Affiliations:** Department of Gastroenterology, Union Hospital, Tongji Medical College, Huazhong University of Science and Technology, Wuhan, People’s Republic of China

**Keywords:** chronic pancreatitis, gut microbiome, meta-analysis, pancreatic cancer

## Abstract

**Background:**

The study of changes in the microbiome in chronic pancreatitis (CP) and pancreatic ductal adenocarcinoma (PDAC) holds significant potential for developing noninvasive diagnostic tools as well as innovative interventions to alter the progression of diseases. This systematic review and meta-analysis aimed to analyze in detail the taxonomic and functional characteristics of the gut microbiome in patients with CP and PDAC.

**Methods:**

Two researchers conducted a systematic search across public databases to gather all published research up to June 2023. Diversity and gut microbiota composition are the main outcomes the authors focus on.

**Results:**

This meta-analysis included 14 studies, involving a total of 1511 individuals in the PDAC (*n*=285), CP (*n*=342), and control (*n*=649) groups. Our results show a significant difference in the composition of gut microbiota between PDAC/CP patients compared to healthy controls (HC), as evidenced by a slight decrease in α-diversity, including Shannon (SMD=−0.33; *P*=0.002 and SMD=−0.59; *P*<0.001, respectively) and a statistically significant β-diversity (*P*<0.05). The pooled results showed that at the phylum level, the proportion of Firmicutes was lower in PDAC and CP patients than in HC patients. At the genus level, more than two studies demonstrated that four genera were significantly increased in PDAC patients compared to HC (e.g. *Escherichia-Shigella* and *Veillonella*). CP patients had an increase in four genera (e.g. *Escherichia-Shigella* and *Klebsiella*) and a decrease in eight genera (e.g. *Coprococcus* and *Bifidobacterium*) compared to HC. Functional/metabolomics results from various studies also showed differences between PDAC/CP patients and HC. In addition, this study found no significant differences in gut microbiota between PDAC and CP patients.

**Conclusions:**

Current evidence suggests changes in gut microbiota is associated with PDAC/CP, commonly reflected by a reduction in beneficial species and an increase in the pathogenic species. Further studies are needed to confirm these findings and explore therapeutic possibilities.

## Graphical abstract

**Figure:**
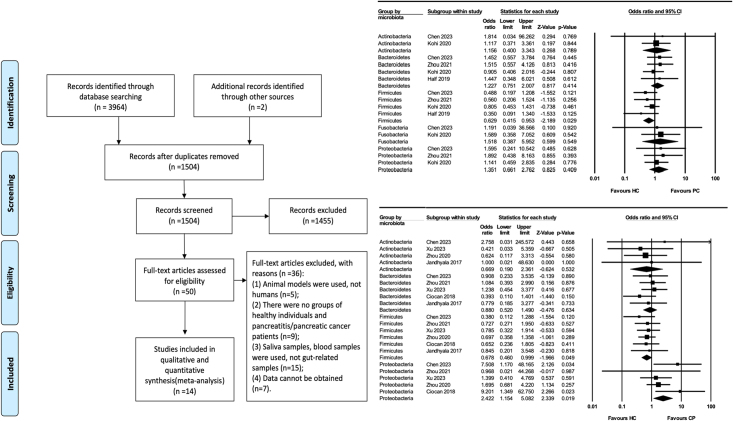

## Introduction

Highlights

- This is the first systematic review and meta-analysis to focus specifically on the gut microbiota of patients with chronic pancreatitis (CP) and pancreatic cancer (PC).
- Compared to the healthy controls, significant differences in gut microbial composition between PC/CP patients, as evidenced by a somewhat reduced α-diversity and statistically significant β-diversity.
- At the phylum level, the proportion of Firmicutes was less abundant in patients with PC and CP than in healthy controls. At the genus level, changes in gut microbiota associated with PC/CP, are commonly reflected by a reduction in beneficial species and an increase in the pathogenic species.

The human microbiota is a leading field of dynamic research in our time, with a predominant focus on the distal gastrointestinal tract, which harbors most of our microbes^1^. The human gut is home to a vast community of 100 trillion microorganisms encompassing more than 1000 distinct resident bacterial species^2^. In the digestive tract, the predominant bacteria are Firmicutes and Bacteroidetes, which account for ~80–90% of the population^3^. Human physiology is significantly impacted by the gut microbiota, as it influences metabolism, regulates the immune system of mucosal tissues, aids in digestion, and maintains intestinal architecture^4^. Dysbiosis of the gut microbiota is linked to various illnesses, including localized gastrointestinal disorders and metabolic, respiratory, hepatic, and neurological disorders^5^.

Intrinsic diseases of the pancreas, pancreatitis, and pancreatic ductal adenocarcinoma (PDAC), cause significant morbidity and mortality in the population^6^. Previously, it was believed that the normal pancreas did not come into direct contact with the intestinal microbiota and thus did not have its own microbiome. The presence of pancreatic microbiota has been demonstrated in a variety of normal and diseased conditions recently^7–9^. Despite this, the routes of bacterial entry into the pancreas remain controversial^6,10^. Pancreatic diseases, including acute pancreatitis (AP), chronic pancreatitis (CP), and pancreatic cancer (PC), are now identified as having altered gut microbiotas^4,11^. Recently, a systematic review reported that changes in microbial composition were associated with both AP and CP^12^. It has been reported that 5 years after diagnosis, patients with CP have a nearly eight-fold increased risk of developing PDAC, suggesting that CP is a risk factor for PDAC^13^. Therefore, it is significant to clarify the microbiota imbalance in these pancreatic diseases, especially in patients with CP and PDAC.

This systematic review aimed to examine the taxonomic and functional characteristics of the gut microbiome in individuals with CP and PDAC. By summarizing these findings, we can determine the relationship between alterations in the gut microbiota and worsening pancreatitis or driving cancer progression. The study of changes in the microbiome in CP and PDAC holds significant potential for developing noninvasive diagnostic tools as well as innovative interventions to alter the progression of these diseases.

## Method

This study was conducted following the PRISMA (Preferred Reporting Items for Systematic Reviews and Meta-Analyses), MOOSE (Meta-Analysis of Observational Studies in Epidemiology), and AMSTAR (Assessing the methodological quality of systematic reviews) Guidelines (Supplementary Table S1, Supplemental Digital Content 1, http://links.lww.com/JS9/C704, Supplementary Table S2, Supplemental Digital Content 2, http://links.lww.com/JS9/C705)^14–16^. This study was performed using publicly accessible data sourced exclusively from previously approved ethical studies. The research protocol for this study has been duly registered in the International Registry of PROSPERO (CRD42023484881).

### Literature search strategy

An independent and comprehensive search was performed in several public databases, with PubMed, Embase (via OVID), Web of Science, and Cochrane Library by two reviewers. The aim was to identify all original research related to the topic. The following terminologies were used: ‘microbiota’, ‘gut microbiota’, ‘gastrointestinal microbiome’, ‘pancreatic cancer’, ‘pancreatic carcinoma’, ‘pancreatic’, and ‘pancreatitis’. Supplementary Table S3 (Supplemental Digital Content 3, http://links.lww.com/JS9/C706) provides additional details regarding the search strategy employed. The last identification date was June 2023. To identify potential research studies which may have been overlooked during the initial database search, further manual searches were conducted within the relevant literature.

### Inclusion and exclusion criteria

The studies were selected based on the following criteria: (1) the study compared the gut microbiota of healthy people with people suffering from PDAC or CP; (2) observational studies (such as cohort studies, case–control studies, and cross-sectional studies). Exclusion criteria: (1) the samples collected in the study are not gut-related samples, such as blood, oral samples, tissue samples, etc.; (2) animal studies were excluded; (3) the study was presented as a conference abstract, case study, or narrative review; (4) the study was not authored in the English language.

### Data extraction and quality assessment

Two reviewers independently screened the titles and abstracts of the retrieved studies. In case of any discrepancies, they engaged in collaborative discussions to reach a consensus, seeking guidance from a third experienced reviewer whenever necessary. Following are the details extracted: author name, year of publication, country, type of study design, characteristics of participants, type of samples, genetic analysis, and result of the gut microbiome. To assess the methodological quality of the observational studies, the Newcastle–Ottawa Scale (NOS) was applied^17^. A score of 7–9 indicated superior quality.

### Statistical analysis

To address differences in gut microbiota, we conducted a literature review and reported partial meta-analysis results at the phylum level. A descriptive synthesis was conducted since microbiota proportions were assessed differently, as well as the quality of included studies was inadequate. Meta-analysis were carried out using Comprehensive Meta-Analysis 3.0. The Shannon Index, Simpson Index, Evenness, and Richness of bacteria were calculated as standardized mean differences (SMDs) between the PDAC, CP, and control groups^18^. We used the inverse variance method to pool odds ratios (ORs) with corresponding 95% CIs to determine the composition of gut microbiota at the phylum level. For continuous variables where median and interquartile range were provided by studies instead of mean and SD, we utilized Wan *et al*.’s^19^ method to calculate them. Statistical significance was set at *P*<0.05. Heterogeneity was measured using *I* ^2^ statistics for all studies; fixed-effects models were applied when *I* ^2^<50%, while random-effects models were applied when *I* ^2^>50%. In cases where there was significant heterogeneity among included studies in the meta-analysis (*n*>10), funnel plots were employed to observe publication bias or other biases that may have affected results obtained from these sources. Finally, sensitivity analyses involved excluding one study after another sequentially until final results or heterogeneity changed significantly enough for us to take note thereof during our analysis process.

## Result

### Study selection

**Figure 1:**
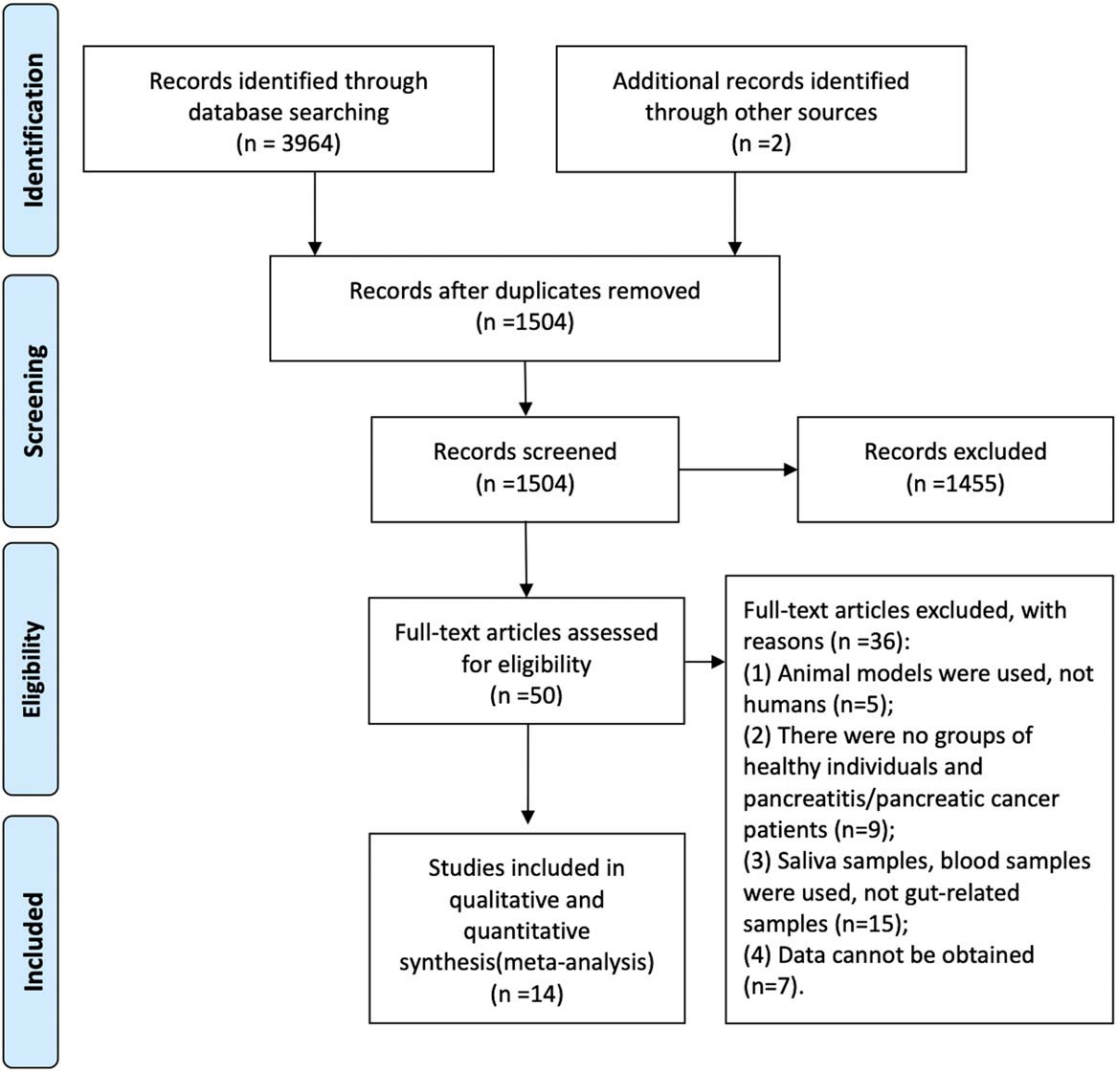
Flow diagram of selection.

A total of 3966 research studies were identified as a result of the systematic literature review. After removing duplicate records, we assessed a total of 1504 records based on their title and abstracts. Subsequently, the search yielded a selection of 50 articles which may have fulfilled all the specified criteria for inclusion. Out of these, 36 studies were excluded due to various reasons such as utilization of animal models instead of human subjects, absence of both healthy individuals and CP/PDAC patients in the study groups, usage of saliva or blood samples rather than gut-related samples, and unavailability of data. The final meta-analysis included 14 studies that adhered strictly to the inclusion and exclusion criteria^11,20–32^. The PRISMA flow chart illustrating this meta-analysis can be observed in Figure 1.

### Study characteristics and quality assessment

Four cohort studies, eight case–control studies, and two cross-sectional studies were included. Three studies reported relevant data in the PDAC and CP groups, four studies reported data only in the PDAC group, and seven studies reported data only in the CP group (compared to healthy individuals). They were published between 2017 and 2023, nine of them from Asia, three from Europe, and two from the United States. A total of 1511 individuals were included, of which 285 were PDAC patients, 342 were CP patients and 649 were healthy individuals. The sample sizes range from 34 to 282. All patients with PDAC are diagnosed by pathology. Patients with CP were diagnosed by guidelines and consensus, with one study reporting that patients with CP had type 1 autoimmune pancreatitis, one study had children with CP, one study had alcoholic pancreatitis, and one study had patients with CP with/without diabetes. The characteristics of the included studies are provided in Table 1. Supplementary Table S4 (Supplemental Digital Content 4, http://links.lww.com/JS9/C707) presents the results obtained from the NOS assessment. The quality of six studies was rated as moderate (7 points), while the quality of eight studies was rated as high (more than 8 points).

**Table 1:** Characteristics of all the studies included in the meta-analysis.

					Number of patients		Sex (male, %)		Age (years)	
Author	Year	Country	Study Design	Participants in experimental group	PDAC /CP	HC	PDAC /CP	HC	PDAC /CP	HC
Chen^20^	2023	China	Case–control	PDAC: pathology; CP: according to the Asia-Pacific consensus	PDAC: 40; CP: 15	39	PDAC: 20 (50.0); CP: 8 (53.3)	20 (51.3)	PDAC: 56.6±9.7; CP: 53.77±11.9	56.4±6.9
Kartal^22^	2022	Spain/Germany	Case–control	PDAC: pathology; CP: clinical symptoms, transsectional imaging and unequivocal evidence	PDAC: 57; CP: 29	50	ND	ND	ND	ND
Zhou^23^	2021	China	Case–control	PDAC: pathology; Type 1 autoimmune CP: according to the consensus by IAP	PDAC: 32; CP: 32	32	PDAC: 25 (78.1); CP: 26 (81.3)	26 (81.3)	PDAC: 59.3±9.5; CP: 58.8±9.8	58.6±10.3
Nagata^11^	2022	Japan	Cohort	PDAC: pathology	PDAC: 47	235	PDAC: 26 (55.3)	130 (55.3)	ND	ND
Hashimoto^21^	2022	Japan	Case–control	PDAC: pathology	PDAC: 5	68	PDAC: 2 (40.0)	29 (42.6)	PDAC: 73.0 (70.0–89.0)	54.0 (50.8–57.3)
Kohi^24^	2020	USA	Case–control	PDAC: pathology	PDAC: 74	134	PDAC: 35 (47.6)	85 (63.5)	PDAC: 63.6 (41.6–79.5)	65.3 (42.2–85.5)
Half^25^	2019	Israel	Cohort	PDAC: pathology	PDAC: 30	13	PDAC: 16 (53.3)	6 (46.2)	PDAC: 68.9±6.2	59.0±8.7
Xu^32^	2023	China	Cross-sectional	CP: according to the Asia-Pacific consensus	CP: 40	38	CP: 25 (62.5)	12 (31.6)	CP: 44±12	49±10
McEachron^31^	2022	USA	Cohort	CP being considered for TPIAT	CP: 20	14	ND	ND	ND	ND
Frost^30^	2020	Germany	Cohort	CP: clinical symptoms, transsectional imaging and unequivocal evidence	CP: 51	102	CP: 40 (78.4)	76 (74.5)	CP: 54.0 (50.0–60.5)	54.0 (43.2–65.0)
Wang^29^	2020	China	Case–control	CP in children: according to the INSPPIRE	CP: 30	35	CP: 16 (53.3)	23 (65.7)	CP: 7.2±0.5	8.3±0.7
Zhou^28^	2020	China	Case–control	CP: according to the Asia-Pacific consensus	CP: 71	69	CP: 41 (57.7)	29 (42.0)	CP: 44±11	47±10
Ciocan^27^	2018	France	Cross-sectional	Alcoholic CP	CP: 24	45	CP: 21 (87.5)	41 (91.1)	CP: 51.5±9.9	51.1±8.5
Jandhyala^26^	2017	India	Case–control	CP with/without diabetes	CP: 30	10	CP: 22 (73.3)	7 (70)	CP: 32.8±10.6	42.3±13.9

CP, Chronic Pancreatitis; HC, Healthy Controls; IAP, International Association of Pancreatology; INSPPIRE, INternational Study Group of Pediatric Pancreatitis: In Search for a CuRE; ND, Not Declared; PDAC, Pancreatic Ductal Adenocarcinoma; TPIAT, Total pancreatectomy with islet autotransplantation.

### Assessment of gut microbiome

The gut microbiome was assessed using duodenal fluid sequencing in one study, and stool samples were used in the remaining 13 studies (Table 2). Among these, metagenomic sequencing was employed in three studies, while one study utilized 18s rRNA gene sequencing. The other studies primarily used Illumina Miseq sequences with 16s rRNA gene sequencing. For pipeline analysis, most researchers relied on Quantitative Insights into Microbial Ecology (QIIME) and Mothur software. Similarly, three main databases – Ribosomal Database Project (RDP) database, Silva database, and Greengenes – were primarily utilized for taxonomic unit assignments from reads.

**Table 2:** Genetic analysis and microbial change (patient vs. control) in the included studies.

Author	Year	Type of samples	Genetic analysis	Microbial change (patient vs. control)	Main functional/metabolomic change (patient vs. control)
Chen^20^	2023	Fecal sample	16s rRNA gene sequencing using Illumina Miseq sequences DNA Extraction: QIAamp Fast DNA Stool Mini Kit (Qiagen) Region: V3-V4 Database: RDP	↑ Proteobacteria(P) and *Peptostreptococcus*, *Actinomyces*, *Bifidobacterium*, *Campylobacter*, *Coprobacillus*, and *Escherichia-Shigella* (G) (CP+PDAC vs. HC); *Prevotella*, *Coprobacter* (G) (PDAC vs. CP+HC)	KEGG analysis: inflammatory pathway activation and decreased cell motility (LPS biosynthesis and peptidoglycan biosynthesis were enhanced, cytoskeleton biosynthesis was weakened) (PDAC vs. HC)
Kartal^22^	2022	Fecal sample	Metagenomics and 16s rRNA gene sequencing using Illumina Miseq and Illumina HiSeq 4000 sequences DNA Extraction: Qiagen DNeasy blood and tissue kit Region: V4	↑ *Veillonella atypica*, *Fusobacterium nucleatum/hwasookii*, and *Alloscardovia omnicolens* (S) (PDAC vs. HC); ↓ *Romboutsia timonensis*, *Faecalibacterium prausnitzii*, *Bacteroides coprocola*, and *Bifidobacterium bifidum* (S) (PDAC vs. HC)	ND
Zhou^23^	2021	Fecal sample	Metagenomics sequencing using Illumina Hiseq sequences DNA Extraction: QIAampPowerFecal Pro DNA Kit	↑ Proteobacteria, Fusobacteria (P) and *Veillonella*, *Escherichia* (G) and *Escherichia coli*, *Fusobacterium nucleatum*, *Clostridium spp, Megamonas spp*, *Veillonella atypica*, *Veillonella parvula* and *Prevotella stercorea* (S) (PDAC vs. HC); *Veillonella* (G) and *Megamonas spp*, *Veillonella atypica*, *Veillonella parvula* and *Prevotella stercorea* (S) (CP vs. HC); ↓ Firmicutes (P) and *Megamonas*, *Faecalibacterium*, *Eubacterium* and *Coprococcus* (G) and *Faecalibacterium prausnitzii*, *Eubacterium rectale*, *Roseburia intestinalis*, and *Ruminococcussp 5_1_39BFAA* (PDAC vs. HC); *Megamonas*, *Faecalibacterium* (G) and *Faecalibacterium prausnitzii* (S) (CP vs. HC)	KEGG analysis: higher potential to degrade fatty acids and a notably lower metabolic capacity to synthesize SCFAs, especially acetate and butyrate; higher potential for putrescine and spermidine transportation; increased T2S pathway, T6S, LPS biosynthesis and upregulated M00210 which can increase gram-negative bacterial vitality through contributing to asymmetric lipid distribution (PDAC vs. HC); increased T2S, M00335, M00210 and decreased T4S, but no significant changes were observed in SCFA and polyamine production (CP vs. HC)
Nagata^11^	2022	Fecal sample	Metagenomics sequencing using Illumina Hiseq X sequences	↑ *Streptococcus anginosus, Clostridium symbiosum, unknown Mogibacterium, Streptococcus oralis, Clostridium clostridioforme, Veillonella atypica, Streptococcus vestibularis, Sutterella wadsworthensis, Actinomyces sp. ICM39, Veillonella parvula, Clostridium boltae/clostridioforme, Erysipelotrichaceae sp., Hungatella hathewayi, unknown cellular organisms, Anaerotruncus colihominis,* and *Streptococcus sp. HSISM1* (S) (PDAC vs. HC) ↓ *Eubacterium sp. CAG:156, Bacteroidetes Blautia wexlerae, Ruminococcus bromii, Bacteroides rodentium/uniformis, Faecalibacterium prausnitzii* *unknown Clostridiales, Eubacterium eligens, Ruminococcus bicirculans, Faecalibacterium prausnitzii, unknown Butyricicoccus, unknown Lachnospiraceae, Eubacterium sp. CAG:38, unknown Clostridiales,* and *Eubacterium ventriosum* (S) (PDAC vs. HC)	KEGG analysis: enriched in the phosphotransferase systems, ABC transporters, and terpenoid backbone biosynthesis; and depleted in amino acid and secondary metabolite biosynthesis and in porphyrin and chlorophyll metabolism
Hashimoto^21^	2022	Fecal sample	16s rRNA gene sequencing using Illumina Miseq sequences DNA Extraction: GENE PREP STAR PI-480 Region: V1-V2 Pipeline analysis: QIIME2 Database: SILVA v132	↑ *Actinomyces*, *Lactobacillus*, *Streptococcus*, and *Veillonella* (G) (PDAC vs. HC); ↓ *Anaerostipes* (G) (PDAC vs. HC)	Metabolomic analysis: a significant decrease in propionic acid and deoxycholic acid, organic acid and bile acid levels remained unchanged (PDAC vs. HC)
Kohi^24^	2020	Duodenal fluid	16s and 18s rRNA gene sequencing using Illumina Miseq sequences DNA Extraction: GENE PREP STAR PI- 480 Region: V3-V4 (16s rRNA) and ITS1 (18s rRNA) Pipeline analysis: QIIME2 Database: SILVA v132 (bacteria) and UNITE v7.2 (fungi)	↑ Fusobacteria, Asomycota (P) and *Fusobacterium*, *Enterococcus*, *Bifidobacterium*, and *Nakaseomyces* (G) (PDAC vs. HC)	ND
Half^25^	2019	Fecal sample	16s rRNA gene sequencing using Illumina Miseq sequences DNA Extraction: PowerSoilTM DNA extraction kit (MOBIO) Pipeline analysis: QIIME Database: RDP and SILVA v128	↑ *Megasphaera*, *Lachnospiraceae UCG_008*, *Akkermansia* (G) (PDAC vs. HC); ↓ *Clostridium sensu stricto 1* (G) (PDAC vs. HC)	ND
Xu^32^	2023	Fecal sample	16s rRNA gene sequencing using Illumina Miseq sequences DNA Extraction: Fast DNA SPIN Extraction Kit (MP) Region: V3-V4 Pipeline analysis: QIIME Database: RDP; SILVA v128	↓ Actinobacteria, Chloroflexi, Acidobacteria, Saccharibacteria, Cyanobacteria, Gemmatimonadetes, and Planctomycetes(P), and *Bifidobacterium* (G) (CP vs. HC)	Metabolomic analysis: the abundances and concentrations of palmitoleic acid, isovaleric acid, 3-methylindole, phenylacetic acid, valeric acid, 5-dodecenoic acid, and caproic acid were all significantly higher, while those of oxoglutaric acid, citric acid, oxoadipic acid, and 3-methyl-2-oxovaleric acid were all significantly lower (CP vs. HC)
McEachron^31^	2022	Fecal sample	16s rRNA gene sequencing using Illumina Miseq sequences DNA Extraction: DNeasy PowerSoil DNA isolation kit (Qiagen) Region: V4 Pipeline analysis: Mothur 1.41.1 Database: RDP	↑ *Bacteroides* and *Escherichia-Shigella* (G) (CP vs. HC); ↓ *Faecalibacterium*, *Coprococcus* and *Clostridium XVIII* (G) (CP vs. HC)	ND
Frost^30^	2020	Fecal sample	16s rRNA gene sequencing using Illumina Miseq sequences DNA Extraction: PSP Spin Stool DNA Kit (AG) Region: V1-V2 Database: RDP	↑ *Bacteroides, Clostridium XlVa, Escherichia-Shigella, Butyricimonas, Streptococcus, Flavonifractor, Clostridium XVIII,* and *Enterococcus* (G) (CP vs. HC); ↓ *Prevotella, Faecalibacterium, Parasutterella, Holdemanella, Alloprevotella, Coprococcus, Clostridium IV, Anaerotruncus, Paraprevotella, Fusicatenibacter, Catenibacterium, Catabacter, Desulfovibrio, Coprobacter,* and *Olsenella* (G) (CP vs. HC)	ND
Wang^29^	2020	Fecal sample	16s rRNA gene sequencing using Illumina Miseq sequences DNA Extraction: OMEGA-soil DNA Kit (Omega BioTek) Region: V3-V4 Database: RDP; SILVA v123	↓ *Propionibacterium, Rhodococcus, Alloprevotella, Actinomyces, Lactobacillus, Enterobacter, Streptococcus, Klebsiella,* and *Enterococcus* (G) (CP vs. HC)； ↑*Faecalibacterium, Bifidobacterium, Eubacterium, Subdoligranulum, Collinsella, Phascolarctobacterium, Roseburia, Fusicatenibacter, Lachnospiraceae, Ruminococcaceae, Haemophilus, Ruminiclostridium, Butyricicoccus, Parasutterella, Erysipelotrichaceae, Lachnospira, Flavonifractor, Actinobacillus,* and *Holdemania* (G) (CP vs. HC)	KEGG analysis: enriched in the phosphotransferase system and depleted in ribosomal activity, porphyrin and chlorophyll metabolism, starch and sucrose metabolism, and aminoacyl-tRNA biosynthesis (CP vs. HC)
Zhou^28^	2020	Fecal sample	16s rRNA gene sequencing using Illumina Miseq sequences DNA Extraction: Fast DNA SPIN Extraction Kit (MP) Region: V3-V4 Pipeline analysis: QIIME Database: RDP; SILVA v128	↑ Proteobacteria (P) and *Escherichia-Shigella*, *Parabacteroides* and *Prevotella* (G) (CP vs. HC); ↓ Actinobacteria and Firmicutes (P) and *Faecalibacterium* and *Subdoligranulum* (G) (CP vs. HC)	KEGG analysis: the pathways of LPS biosynthesis and bacterial invasion of epithelial cells were enriched, arginine and proline metabolism and the glycolysis/gluconeogenesis pathways were highly depleted (CP vs. HC)
Ciocan^27^	2018	Fecal sample	16s rRNA gene sequencing using Illumina Miseq sequences DNA Extraction: guanidinium thiocyanate lysis buffer Region: V3-V4 Pipeline analysis: QIIME Database: Greengenes 13.8	↑ Proteobacteria (P), *Aquabacterium*, *Enterococcus*, *Klebsiella*, *Pseudomonas* and *Sphingomonas* (G) (CP vs. HC); ↓ Bacteroidetes and Fusobacteria (P), *Anaerostipes*, *Bacteroides*, *Bilophila*, *Lactococcus*, *Paraprevotella*, *Roseburia* and *Sutterella* (G) (CP vs. HC);	ND
Jandhyala^26^	2017	Fecal sample	16s rRNA gene sequencing using Illumina Miseq sequences DNA Extraction: QIAGEN mini stool DNA isolation kit Region: V3-V4 Database: RDP	↑ ratio (Firmicutes : Actinobacteria) (P) (CP vs. HC); ↓ Bacteroidetes (P), *Faecalibacterium prausnitzii* and *Ruminococcus bromii* (S) (CP vs. HC); ↔ Class, order, family levels, *Faecalibacterium* (G) (CP vs. HC)	KEGG analysis: significant increase in LPS synthetic pathways, and increase in the plasma endotoxin levels

ABC, ATP-binding cassette; CP, chronic pancreatitis; HC, healthy controls; KEGG, kyoto encyclopedia of genes and genomes; LEfSe, linear discriminant analysis (LDA) effect size; LPS, lipopolysaccharide; ND, not declared; PDAC, pancreatic ductal adenocarcinoma; SCFAs, short-chain fatty acids; T2S, type II general secretion pathway; T6S, type VI secretion system.

### Summary and meta-analysis of alpha-diversity outcomes

In terms of alpha-diversity outcomes, all 14 studies employed seven different types of assessments. The Shannon Index and Simpson Index were frequently measured among these assessments. A comprehensive summary of α- diversity analysis can be found in Supplementary Table S5 (Supplemental Digital Content 5, http://links.lww.com/JS9/C708).

Five studies have reported results comparing the PDAC group with the healthy controls (HC) group. The pooled results showed that the PDAC group had a significantly lower Shannon Index (SMD=−0.33; 95% CI: −0.53 to −0.12; *P*=0.002) and Evenness (SMD=−0.33; 95% CI: −0.63 to −0.02; *P*=0.037), while the Simpson Index (SMD=−0.30; 95% CI: −0.61 to 0.00; *P*=0.052) and Richness (SMD=−0.07; 95% CI: −0.32 to 0.18; *P*=0.583) had no significant difference (Table 3 and Supplementary Fig S1, Supplemental Digital Content 6, http://links.lww.com/JS9/C709).

**Table 3:** The meta-analyses of alpha-diversity outcomes of the included studies.

Comparison	No. of study	SMD	95% CI	*Z* value	*P*
PDAC vs. HC					
Shannon Index	4	−0.325	[−0.529 to −0.121]	−3.122	0.002
Simpson Index	2	−0.303	[−0.609–0.002]	−1.945	0.052
Evenness	2	−0.326	[−0.633 to −0.019]	−2.083	0.037
Richness	3	−0.070	[−0.319–0.179]	−0.548	0.583
CP vs. HC					
Shannon Index	9	−0.593	[−0.747 to −0.439]	−7.544	<0.001
Simpson Index	6	−0.113	[−0.282–0.056]	−1.315	0.189
Evenness	2	−0.244	[−0.580–0.092]	−1.423	0.155
Richness	6	−0.426	[−0.619 to −0.233]	−4.331	<0.001
CP vs. PDAC					
Shannon Index	2	0.069	[−0.266–0.404]	0.406	0.685
Simpson Index	2	0.037	[−0.297–0.371]	0.218	0.827
Evenness	2	0.038	[−0.296–0.371]	0.221	0.825
Richness	3	−0.103	[−0.394–0.188]	−0.694	0.488

CP, chronic pancreatitis; HC, healthy controls; OR, odds ratio; PDAC, pancreatic ductal adenocarcinoma; SMD, standardized mean difference.

A total of 10 studies reported results comparing α-diversity in the CP group with the HC group. The pooled results showed that the CP group had a significantly lower Shannon Index (SMD=−0.59; 95% CI: −0.75 to −0.44; *P*<0.001) and Richness (SMD=−0.43; 95% CI: −0.62 to −0.23; *P*<0.001), while the Simpson Index (SMD=−0.11; 95% CI: −0.28 to 0.06; *P*=0.189) and Evenness (SMD=0.24; 95% CI: −0.58 to 0.09; *P*=0.155) had no significant difference (Table 3 and Supplementary Fig S1, Supplemental Digital Content 6, http://links.lww.com/JS9/C709).

Only three studies reported results comparing the CP group with the HC group. Between patients with CP and PDAC, Shannon Index, Simpson Index, Evenness, and Richness did not differ significantly (*P*>0.05). (Table 3 and Supplementary Fig S1, Supplemental Digital Content 6, http://links.lww.com/JS9/C709).

### Summary of beta-diversity outcomes

β-diversity results were reported in all 14 studies, which used four different types of β-diversity assessment methods. Of these assessments, principal coordinate analysis (PCoA) is the most commonly measured. A detailed sum-up of the β diversity analysis is shown in Supplementary Table S5 (Supplemental Digital Content 5, http://links.lww.com/JS9/C708).

Seven studies compared the PDAC and HC groups, six of which showed statistically significant differences in gut microbiome composition between them and one showed no significant differences.

Of the nine studies that reported CP versus HC groups, six had statistically significant differences in the intestinal microbiota composition between them, and one had no significant differences. The remaining two items had no significant difference at PCoA of weighted UniFrac distances but had statistical differences at PCoA of unweighted UniFrac distances.

Three studies reported the comparison of β-diversity between the CP group and PDAC group, and all three studies showed statistically significant differences in the composition of gut microbial community between PDAC and CP.

### Summary and meta-analysis of gut microbiota composition

All 14 studies reported significant microbiota changes in patients with PDAC/CP. We summarized the classification of microorganisms that differed significantly at various levels (e.g. phylum, genus, and species level) in PDAC/CP patients compared to HC (Table 2).

**Figure 2:**
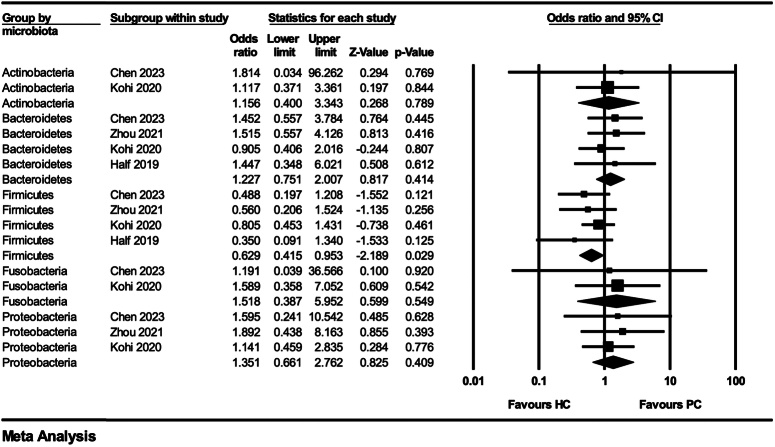
Forest plot of meta-analysis of the composition of gut microbiota at phylum level (pancreatic cancer versus healthy individuals).

Compared to HC, the gut microbiota composition in PDAC patients is different. At the phylum level, more than two studies presented an increase in the proportion of Proteobacteria and Fusobacterium phylum in patients with PDAC. At the genus level, 22 genera were identified in five studies. The following results were found in more than two studies with the same trend: *Actinomyces*, *Bifidobacterium*, *Veillonella*, and *Escherichia-Shigella* were increased in patients with PDAC compared to HC. At the phylum level, we conducted a meta-analysis of gut microbiota, but the pooled results showed that compared to the HC group, the proportion of Firmicutes in PDAC patients was significantly reduced (OR=0.63; 95% CI: 0.42–0.95; *P*=0.029; *I* ^2^=0%), but there was no significant difference in other phyla (Actinobacteria, Bacteroidetes, Fusobacteria, and Proteobacteria) (*P*>0.05) (Fig. 2).

**Figure 3:**
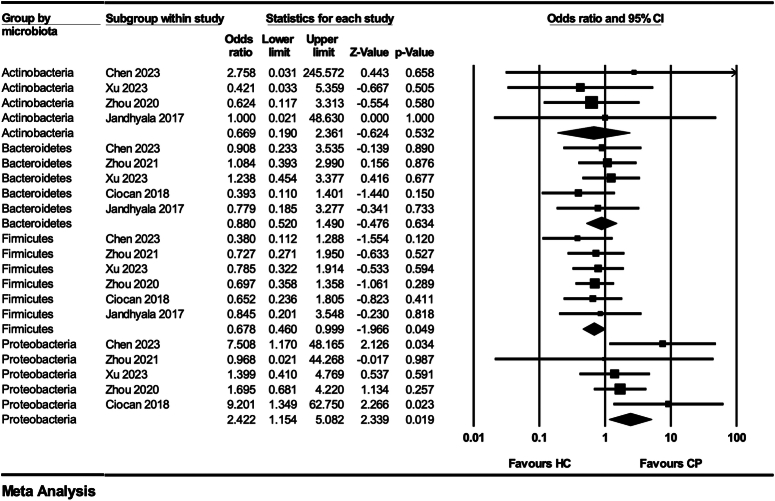
Forest plot of meta-analysis of the composition of gut microbiota at phylum level (chronic pancreatitis versus healthy individuals).

There were also differences in the gut microbiota of patients in the CP group compared to the HC group. At the phylum level, more than two studies reported an increase in the proportion of Proteobacteria and a decrease in the proportion of Bacteroidetes and Actinobacteria in patients with CP. At the genus level, 52 genera were identified in seven studies. The following results were presented in more than two studies with the same trend: *Escherichia-Shigella*, *Enterococcus*, *Klebsiella*, and *Streptococcus* were increased in CP patients, while *Faecalibacterium*, *Bifidobacterium*, *Coprococcus*, *Subdoligranulum*, *Parasutterella*, *Paraprevotella*, *Fusicatenibacter*, and *Roseburia* and were decreased compared to HC. At the phylum level, we conducted a meta-analysis, and the pooled results showed a significantly lower proportion of Firmicutes in patients with CP compared to the HC group (OR=0.68; 95% CI: 0.46–0.99; *P*=0.049; *I* ^2^=0%), but there was no significant difference in other phyla (Actinobacteria, Bacteroidetes, and Proteobacteria) (*P*>0.05) (Fig. 3).

**Figure 4:**
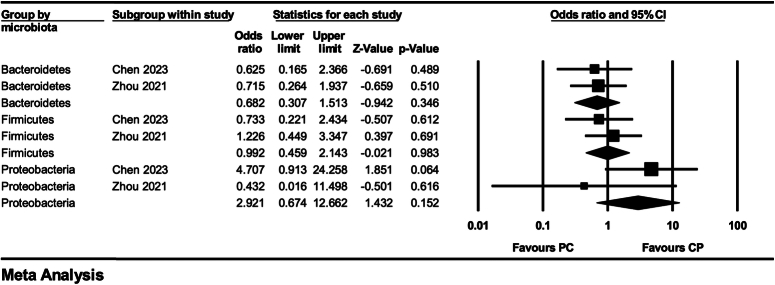
Forest plot of meta-analysis of the composition of gut microbiota at the phylum level (chronic pancreatitis versus pancreatic cancer).

In addition, two studies reported differences in gut microbiota at the phylum level between the PDAC and CP groups, and our pooled results showed no significant difference in the proportions of Bacteroidetes, Firmicutes, and Proteobacteria between the CP and PDAC groups (*P*>0.05) (Fig. 4). Likewise, at the genus level, no more than two studies reported significant changes in the microbiota with the same trend.

### Summary of functional/metabolomic change

Compared to the HC group, the PDAC group had a significant increase in lipopolysaccharide (LPS) synthesis^20,23^, phosphotransferase systems, ATP-binding cassette transporters, and terpenoid backbone biosynthesis^11^, activation of inflammatory pathways, and decreased cell motility^20^. In terms of metabolic pathways, the PDAC group had a higher potential for degrading fatty acids, a significantly lower metabolic capacity for the synthesis of short-chain fatty acids (SCFA), a higher potential for putrescine and spermidine transport^23^, and a significant decrease in propionic acid and deoxycholic acid^21^, amino acid, secondary metabolite biosynthesis, and in porphyrin and chlorophyll metabolism^11^.

Similarly, the LPS synthesis pathway^26,28^ and bacterial invasion pathway^28^ were enriched in the CP group compared to the HC, increasing plasma endotoxin levels^26^. Additionally, the phosphotransferase system of the CP group was enriched while ribosomal activity was reduced^29^. In terms of metabolism pathways, chlorophyll metabolism, and porphyrin, sucrose and starch metabolism are depleted^29^, arginine and proline metabolism, as well as glycolysis and gluconeogenesis pathways, are severely depleted^28^.

### Publication bias and sensitivity analysis

A 0% *I* ^2^ value indicates minimal heterogeneity among studies of gut microbiota composition between PDAC/CP and HC groups. The heterogeneity of the combined results of the meta-analysis is shown in detail in Supplementary Table S6 (Supplemental Digital Content 7, http://links.lww.com/JS9/C710). Funnel charts should not be used as the number of included studies for each indicator did not exceed ten. To assess if a single research would affect the overall results, we used sensitivity analysis. After each study was deleted individually, the analysis was largely stable (Supplementary Fig. S2, Supplemental Digital Content 8, http://links.lww.com/JS9/C711).

## Discussion

In the past, several reviews have explored the role of the human microbiome in pancreatic disease^12,33,34^, but to our knowledge, this is the first systematic review to focus specifically on the gut microbiota of patients with CP and PDAC. In this meta-analysis of 14 studies, we systematically assessed the correlation between gut microbiota and PDAC/CP. First, we found differences in the composition of gut microbiota between PDAC/CP patients compared to the HC, as evidenced by a somewhat reduced α-diversity and statistically significant β-diversity. Second, at the phylum level, the proportion of Firmicutes was less abundant in PDAC and CP patients than in HC. Third, at the genus level, *Actinomyces*, *Bifidobacterium*, *Veillonella*, and *Escherichia-Shigella* were increased in PDAC patients compared to HC. *Streptococcus*, *Klebsiella*, *Enterococcus*, and *Escherichia-Shigella* were increased in CP patients, while *Faecalibacterium*, *Bifidobacterium, Coprococcus*, *Subdoligranulum*, *Fusicatenibacte*r, *Roseburia*, *Parasutterella*, and *Paraprevotella* were decreased compared to HC. Finally, functional/metabolomics results from various studies also showed differences between patients with PDAC/CP and the HC. Additionally, our study finds no significant differences in intestinal microbiota between PDAC patients and CP patients.

While the diversity metrics themselves may not have enough resolution to serve as diagnostic or prognostic markers, analyzing diversity is a convincing tool for broadly evaluating the composition of the gut microbiota. It offers a less biased approach compared to culture-dependent or limited targeted assay methods^12,35^. Regarding diversity assessment, to our knowledge, none of the previous studies have performed meta-analyses of α and β diversity assessments in PDAC and CP patients. Our meta-analysis revealed that both the PDAC group and the CP group had lower Shannon index values compared to the HC group, with no significant difference between the PDAC and CP groups. The Shannon index primarily considers two factors: the number of species as operational taxonomic units and their relative abundance inequality^36,37^. However, due to the fact that each study includes a different number of species, the Shannon index does not fully assess inequality. One study showed that long-surviving PDAC patients have higher α-diversity in their tumor microbiome, and it has been demonstrated through fecal microbiome transplantation (FMT) experiments that the composition of the microbiome in PDAC that cross-talks to the gut microbiome, influences the host immune response and natural history of the disease^38^. Regarding β-diversity analysis, we were unable to conduct a meta-analysis due to insufficient data; therefore we can only describe the results of individual studies in detail. We found that most studies reported significant differences between the PDAC/CP and HC groups. Also, interestingly, there were differences between the PDAC and CP groups. It has been shown in multiple studies that CP disease severity is linked to significant changes in the composition of gut microbiome and reduced relative abundance of commensal organisms^26,28^. Regardless of the underlying cause of CP, similar discoveries have been documented^23,39^, suggesting that changes in the microbial composition may be an effect rather than a cause of CP^12^. Therefore, there is a clear need to further investigate the differences in microbial species diversity among PDAC, CP, and HC patients.

The abundance of Firmicutes was decreased in the gut of PDAC patients and CP compared to HC patients, based on pooled results. Firmicutes consist of a large number of core bacteria with more diverse functions. From a metabolic point of view, these bacteria play a concernful role in host and gut homeostasis through the production of SCFA, which normalizes intestinal permeability. An increase or decrease in the Firmicutes/Bacteroidetes ratio is considered an ecological imbalance^40^. The former is often associated with metabolic disorders and obesity. The latter is associated with depression, inflammatory bowel disease, etc., which may be related to the immunoinflammatory response induced by the reduced production of SCFA, especially butyric acid, histamine, LPS accumulation, etc.^41^. *Faecalibacterium* and *Coprococcu* are among the most abundant commensal bacteria in the intestine and are members of the Firmicutes phylum. In addition, they are an important source of butyric acid production in their gut microbiota. Multiple studies have consistently presented a clear reduction in butyric-producing bacteria in CP patients, such as *Faecalibacterium*, *Coprococcu*, *Roseburia,* and *Fusicatenibacter*. The decrease of butyric acid, butyrate, and other strains reduces the levels of tight junction protein and SCFA, increases the permeability of human intestinal mucosa and the dysfunction of the intestinal mucosal barrier, resulting in the release of intestinal pathogens into the blood through the damaged intestinal mucosa, thereby promoting the occurrence of pancreatitis^42^. At present, we can only speculate that there may be a potential association with the occurrence of pancreatic inflammation, but the specific causal relationship needs to be further demonstrated.

*Bifidobacteria* are anaerobic bacteria and are considered to be an important beneficial microbe in the gut. *Bifidobacterium* is often used in combination with *Lactobacillus* to treat chronic diarrhea and constipation and to assist in the treatment of endotoxemia caused by intestinal microbiota imbalance. One study reported a lower abundance of *Bifidobacterium* in the intestinal of PDAC patients compared to HC^22^, whereas one studies reported a higher abundance of *Bifidobacterium* in PDAC patients (duodenal fluid samples)^24^. Correspondingly, studies have reported that repopulation of the germ-free KC genetic mouse model of pancreatic tumors, with *Bifidobacterium pseudopodium*-accelerated pancreatic tumorigenesis and can be detected in the pancreas of treated mice^9^. However, many recent studies have found that *Bifidobacterium* strains enhance the efficacy of anti-PD-L1 treatment and synergistically reduce tumor burden by inducing antitumor host immune response^43–45^. These studies and findings contribute to the application of strategies to modulate the intestinal microbiota to cancer immunotherapy in clinical practice, providing evidence for the treatment of PDAC with live microbial products. Two studies reported lower abundances of *Bifidobacterium* in the intestinal of CP patients compared with HC^29,32^. In a recent study, fecal *Bifidobacteria* abundance was found to be inversely correlated with systemic inflammation in patients with pancreatitis. It has also been found that *Bifidobacteria* and its metabolite lactate prevent pancreatitis in mice by alleviating macrophage-associated pancreatic and systemic inflammation through the combined action of TLR4/MyD88 and NLRP3/Caspase1^46^.

Several studies have reported significantly higher abundances of *Escherichia-Shigella* in the intestines of both PDAC and CP patients compared to HC^20,23,30,31^. *Escherichia-Shigella* is the most common pathogen of bacterial dysentery in humans, producing endotoxins and exotoxins that can damage the intestinal mucosa. Bacterial invasion of epithelial cells in pancreatitis has been reported, highly correlated with the abundance of *Escherichia-Shigella* ^47^. In addition, studies have found significant changes in the gut microbiota of mice with or without pancreatitis after NLRP3 knockout, suggesting that NLRP3 deficiency can counteract pancreatitis-induced microbial interference^48^. Several studies have reported an increase in *Klebsiella* in the intestinal of CP patients compared to HC^27,29^. *Klebsiella* is significantly elevated in pancreatic cysts or pancreatitis fluid, suggesting that the intestine is likely to be the pathway for *Klebsiella* to invade the pancreas^49,50^.

Establishing a distinct correlation between alterations in the gut microbiota and pancreatitis/PDAC holds significant significance, given its potential to offer valuable avenues for clinical intervention. However, based on the current evidence, we cannot investigate whether dysbiosis is pathogenic or reactive. Therefore, it is also indispensable to explore the pathogenic mechanism, for example, intestinal barrier dysfunction, bacterial translocation, microcirculation dysfunction and autodigestion may also be related to pancreatic inflammation^51,52^. Further studies are ongoing, for example, in PROCEED, immunoassay results are reported for serum samples collected from large pancreatitis cohorts, identifying immune markers that could act as potential biomarkers to distinguish different pancreatitis diseases^53^. However, the results of whether CP and diabetes alter the microbiome and lead to PDAC have not been published^54^. A recent two-sample Mendelian Randomization (MR) analysis to investigate the causal relationship effect between intestinal microbiota and PDAC showed that *Senegalimassilia* showed protective effects against PDAC, while *Streptococcus*, *Odoribacter*, *Ruminiclostridium 9*, and *Ruminococcaceae (UCG011)* were identified as causative factors for PDAC^55^. Another MR study involving 18 340 participants in a genome-wide association study (GWAS) explored the causal effect between intestinal microbiota and four types of pancreatitis (acute and CP and alcohol-induced acute and CP), revealing correlations between pancreatitis and 30 different types of intestinal microbiota^56^. Identifying these potential associations will be important for preventing and treating pancreatic disease, as well as determining how to apply strategies that alter the gut microbiota, for example, antibiotics, probiotics, dietary changes, and FMT.

This systematic review and meta-analysis has several limitations. First, the selected studies had a wide range of participants. Several of the included studies involved subjects with specific types of pancreatitis, including autoimmune pancreatitis, alcoholic pancreatitis, childhood pancreatitis, and pancreatitis with diabetes^23,26,27,29^. Age, alcohol, metabolism, and immunity may affect the distribution of microbiota^57^. Second, different methods of genetic analysis were used in the included studies, including sequencing methods, regional specifications, pipeline analyses, and database. It is possible that the results were influenced by inconsistencies in methodology, specifically in specimen handling, DNA extraction protocols, and sequencing methods. Third, although the included studies were conducted on a global scale, there was not enough information to allow us to perform a subgroup analysis comparing different geographical zones. Due to dietary differences between countries, there are conspicuous differences in the composition of nutrients among Asians, Americans, and Europeans, which will have a considerable influence on the composition of intestinal microbiota. Fourth, though our study found correlations between the gut microbiome and PDAC/CP, the observational nature of the study makes it difficult to determine a causal relationship between the gut microbiome and PC. Fifth, the number of studies examining the relationship between PDAC and CP is small, so it is not possible to produce more reliable results. Sixth, several studies presented taxonomic data at different taxonomic levels as well as reported species-level data obtained using Metagenomics sequencing.

## Conclusion

In conclusion, current evidence suggests changes in gut microbiota associated with PDAC/CP, commonly reflected by a reduction in beneficial species and an increase in the pathogenic species. The difference between PDAC and CP has not been found. Functional/metabolomic results from various studies also show differences between patients with pancreatic disease and HC. Detection of differences in microbial composition associated with CP and PDAC could serve as a biomarker and be a diagnostic tool. However, further research is needed to elucidate the potential role of the gut microbiome in pancreatic disease, which may provide evidence for biomarkers and microbial therapies.

## Ethical approval

Not applicable (this paper was provided based on research in global databases).

## Consent

Not applicable (this paper was provided based on research in global databases).

## Source of funding

This work was supported by the National Key Research and Development Program of China (Nos. 2023YFC2307000), National Natural Science Foundation of China (Nos. 82300616, 81974068 and 81770539) and Natural Science Foundation of Hubei (Nos.2023AFB301). The funders had no role in the design of the study, data collection and analysis, interpretation of data, and writing the manuscript.

## Author contribution

Each author contributed significantly to the conception and development of the present paper. R.L. and M.F.: designed the research process; J.H., Y.F., and X.C.: searched the database for corresponding articles and extracted useful information from the articles above; Y.Z., X.L., T.L., and Y.L.: used statistical software for analysis; J.H. and Y.F.: drafted the manuscript; R.L. and M.F.: supervised, reviewed, and revised the manuscript. All authors have read and approved the final version of this manuscript.

## Conflicts of interest disclosure

The authors declare no conflicts of interest.

## Research registration unique identifying number (UIN)

1. Name of the registry: PROSPERO.
2. Unique identifying number or registration ID: CRD42023484881.
3. Hyperlink to your specific registration (must be publicly accessible and will be checked): https://www.crd.york.ac.uk/prospero/display_record.php?ID=CRD42023484881

## Guarantor

The corresponding author, Rong Lin, is the guarantor of this study. Rong Lin, Ph.D. M.D., professor, chief physician, doctoral supervisor, director of the Department of Gastroenterology and Endoscopy Center of Wuhan Union Hospital, ‘Young Yangtze River Scholar’ of the Ministry of Education of China, ‘Outstanding Youth of Hubei Province’, ‘Top Young Talents of Public Health in Hubei Province’, ‘American College of Gastroenterology Jon Isenberg International award’. She has published more than 100 SCI papers in related fields, and has published more than 80 articles as the first author or corresponding author in internationally renowned magazines such as *Gut, Gastroenterology, Endoscopy, Cancer Lett, and Stem Cells*, including five articles with IF>20. Obtained multiple national invention patents and wrote four guides/monographs. As the chief scientist, she presided over 1 national key R&D plan project during the 14th Five-Year Plan, 1 sub-project of the 13th Five-year National Key R&D Plan, 5 National Natural Science Foundation general projects.

## Availability of data and materials

The datasets supporting the conclusions of this article are included within the article. If you want detailed data about this article, please contact the corresponding author.

The data that support the findings of this study are available from the corresponding author, Rong Lin, upon reasonable request.

## Provenance and peer review

Not commissioned, externally peer-reviewed.

## Supplementary Material
