# Supplementary material for: Gut microbiome changes associated with chronic pancreatitis and pancreatic cancer: a systematic review and meta-analysis
Source: Int J Surg. 2024 Jun 7;110(9):5781–94. doi: 10.1097/JS9.0000000000001724 (PMC11392207; doi:10.1097/JS9.0000000000001724)
Supplement: Supplementary file 3 [file js9-110-5781-s003.docx]

**Supplementary Table 3.** Search Strategy.

**Data searched from PubMed**

| Search Query [Title/Abstract] | Results |
| --- | --- |
| #1 probiotics OR prebiotics | 29,239 |
| #2 microbiota OR gut microbiota OR gastrointestinal microbiome OR microflora OR microbiome | 137,075 |
| #3 pancreatic cancer OR pancreatic carcinoma OR pancreatic OR pancreatitis | 226,607 |
| #4 #1 OR #2 | 154,097 |
| #5 #4 AND #3 | 1,256 |

Searched on June 30, 2023. Results: 1,256.

**Data searched from Web of Science**

| Search Query [AB, TI] | Results |
| --- | --- |
| #1 probiotics OR prebiotics | 28,030 |
| #2 microbiota OR gut microbiota OR gastrointestinal microbiome OR microflora OR microbiome | 125,839 |
| #3 pancreatic cancer OR pancreatic carcinoma OR pancreatic OR pancreatitis | 156,295 |
| #4 #1 OR #2 | 143,562 |
| #5 #4 AND #3 | 1,028 |

Searched on June 30, 2023. Results: 1,028.

**Data searched from** **Embase**

| Search Query [ab, ti, kw] | Results |
| --- | --- |
| #1 probiotics OR prebiotics | 40,220 |
| #2 microbiota OR gut microbiota OR gastrointestinal microbiome OR microflora OR microbiome | 153,893 |
| #3 pancreatic cancer OR pancreatic carcinoma OR pancreatic OR pancreatitis | 314,094 |
| #4 #1 OR #2 | 177,681 |
| #5 #4 AND #3 | 1,486 |

Searched on June 30, 2023. Results: 1,486.

**Data searched from Cochrane Library**

| Search Query [ti, ab, kw] | Results |
| --- | --- |
| #1 probiotics OR prebiotics | 10,546 |
| #2 microbiota OR gut microbiota OR gastrointestinal microbiome OR microflora OR microbiome | 11,091 |
| #3 pancreatic cancer OR pancreatic carcinoma OR pancreatic OR pancreatitis | 15,515 |
| #4 #1 OR #2 | 18,342 |
| #5 #4 AND #3 | 194 |

Searched on June 30, 2023. Results: 194.
