# Supplementary material for: Gut microbiome changes associated with chronic pancreatitis and pancreatic cancer: a systematic review and meta-analysis
Source: Int J Surg. 2024 Jun 7;110(9):5781–94. doi: 10.1097/JS9.0000000000001724 (PMC11392207; doi:10.1097/JS9.0000000000001724)
Supplement: Supplementary file 4 [file js9-110-5781-s004.docx]

| Author, year,  Study | **Selection (Out of 4)** | | | | **Comparability**  **(Out of 2)** | **Outcomes (Out of 3)** | | | **Total**  **(Out of 9)** |
| --- | --- | --- | --- | --- | --- | --- | --- | --- | --- |
|  | Representativeness of exposed cohort | Selection of  nonexposed cohort | Ascertainment  of exposure | Outcome not present at the start of the study |  | Assessment of outcomes | Length of follow-up | Adequacy of follow-up |  |
| Chen, 2023 | 1 | 1 | 1 | 0 | 1 | 1 | 1 | 1 | 7 |
| Kartal, 2022 | 1 | 1 | 1 | 1 | 2 | 1 | 1 | 1 | 9 |
| Zhou, 2021 | 1 | 1 | 1 | 1 | 1 | 1 | 1 | 1 | 8 |
| Nagata, 2022 | 1 | 1 | 1 | 1 | 2 | 1 | 1 | 1 | 9 |
| Hashimoto, 2022 | 1 | 1 | 1 | 0 | 1 | 1 | 1 | 1 | 7 |
| Kohi, 2020 | 1 | 1 | 1 | 1 | 1 | 1 | 1 | 1 | 8 |
| Half, 2019 | 1 | 1 | 1 | 0 | 1 | 1 | 1 | 1 | 7 |
| Xu, 2023 | 1 | 1 | 1 | 1 | 1 | 1 | 1 | 1 | 8 |
| McEachron, 2022 | 1 | 1 | 1 | 0 | 1 | 1 | 1 | 1 | 7 |
| Frost, 2020 | 1 | 1 | 1 | 1 | 2 | 1 | 1 | 1 | 9 |
| Wang, 2020 | 1 | 1 | 1 | 1 | 1 | 1 | 1 | 1 | 8 |
| Zhou, 2020 | 1 | 1 | 1 | 1 | 1 | 1 | 1 | 1 | 8 |
| Ciocan, 2018 | 1 | 1 | 1 | 0 | 1 | 1 | 1 | 1 | 7 |
| Jandhyala, 2017 | 1 | 1 | 1 | 0 | 1 | 1 | 1 | 1 | 7 |

**Supplementary Table 4.** Quality assessment of all studies included.

All observational studies were assessed by the Newcastle-Ottawa Quality Assessment Scale.
