# Supplementary material for: Gut microbiome changes associated with chronic pancreatitis and pancreatic cancer: a systematic review and meta-analysis
Source: Int J Surg. 2024 Jun 7;110(9):5781–94. doi: 10.1097/JS9.0000000000001724 (PMC11392207; doi:10.1097/JS9.0000000000001724)
Supplement: Supplementary file 5 [file js9-110-5781-s005.docx]

| **Supplementary Table 5.** **Summary of diversity assessments in the included studies.** | | | | | | |
| --- | --- | --- | --- | --- | --- | --- |
| Study | α diversity | Findings | Statistic value | β diversity | Findings | Statistic value |
| **1. PDAC vs. HC** | | | | | | |
| Chen 2023 | Richness | There was no significant difference between the groups. | *P*=0.95 | PCoA of weighted UniFrac distances | There was a significant difference in gut microbial community composition between the groups. | *P*=0.002 |
|  | Observed species | There was no significant difference between the groups. | *P*=0.84 |  |  |  |
|  | Phylogenetic diversity | There was no significant difference between the groups. | *P*=0.92 |  |  |  |
| Kartal 2022 | Richness | The PDAC group had lower Richness than HC. | *P*=0.05 | RDA based on Bray-Curtis distance | There was a mild but significant difference in gut microbial community composition between the groups. | *P*<0.001 |
|  | Shannon | The PDAC group had lower Shannon diversity than HC. | *P*=0.003 |  |  |  |
|  | Simpson | The PDAC group had lower Simpson diversity than HC. | *P*=0.004 |  |  |  |
|  | Eveness | The PDAC group had lower Eveness than HC. | *P*=0.002 |  |  |  |
| Zhou 2021 | Richness | There was no significant difference between the groups. | *P*=0.26 | PCoA based on Bray-Curtis metrics | There was a significant difference in gut microbial community composition between the groups. | *P*=0.001 |
|  | Shannon | There was no significant difference between the groups. | *P*=0.27 |  |  |  |
|  | Simpson | There was no significant difference between the groups. | *P*=0.23 |  |  |  |
|  | Eveness | There was no significant difference between the groups. | *P*=0.29 |  |  |  |
| Nagata | Shannon | The PDAC group had lower Shannon diversity than HC. | *P*=0.08 | MDS | There was a significant difference in gut microbial community composition between the groups. | *P*<0.001 |
|  | Simpson | There was no significant difference between the groups. | ND |  |  |  |
| Hashimoto 2022 | Shannon | There was no significant difference between the groups. | ND | PCA | There was no significant difference in gut microbial community composition between the groups. | ND |
| Kohi 2020 | Observed species | The PDAC group had lower observed species than HC. | *P*=0.005 | PCoA of unweighted Unifrac distances | There was a significant difference in gut microbial community composition between the groups. | *P*=0.001 |
|  | Shannon | The PDAC group had lower Shannon diversity than HC. | *P*=0.03 |  |  |  |
|  | Phylogenetic diversity | The PDAC group had lower Phylogenetic diversity than HC. | *P*=0.006 |  |  |  |
| Half 2019 | Shannon | There was no significant difference between the groups. | *P*=0.29 | PCoA of weighted UniFrac distances | There was a weak but significant difference in gut microbial community composition between the groups. | *P*=0.013 |
|  |  |  |  | PCoA of unweighted UniFrac distances | There was a weak but significant difference in gut microbial community composition between the groups. | *P*=0.04 |
| **2. CP vs. HC** | | | | | | |
| Chen 2023 | Richness | There was no significant difference between the groups. | *P*=0.23 | PCoA of weighted UniFrac distances | There was a significant difference in gut microbial community composition between the groups. | *P*=0.002 |
|  | Observed species | There was no significant difference between the groups. | *P*=0.19 |  |  |  |
|  | Phylogenetic diversity | There was no significant difference between the groups. | *P*=0.27 |  |  |  |
| Kartal 2022 | Richness | There was no significant difference between the groups. | *P*=0.75 | ND | ND | ND |
|  | Shannon | There was no significant difference between the groups. | *P*=0.035 |  |  |  |
|  | Simpson | There was no significant difference between the groups. | *P*=0.27 |  |  |  |
|  | Eveness | There was no significant difference between the groups. | *P*=0.22 |  |  |  |
| Zhou 2021 | Richness | There was no significant difference between the groups. | *P*=0.51 | PCoA based on Bray-Curtis metrics | There was no significant difference in gut microbial community composition between the groups. | *P*=0.079 |
|  | Shannon | There was no significant difference between the groups. | *P*=0.45 |  |  |  |
|  | Simpson | There was no significant difference between the groups. | *P*=0.39 |  |  |  |
|  | Eveness | There was no significant difference between the groups. | *P*=0.47 |  |  |  |
| Xu 2023 | Observed species | There was no significant difference between the groups. | *P*=0.056 | PCoA of weighted UniFrac distances | There was a significant difference in gut microbial community composition between the groups. | *P*=0.047 |
|  | Shannon | There was no significant difference between the groups. | *P*=0.39 |  |  |  |
|  | Simpson | There was no significant difference between the groups. | *P*=0.69 | PCoA of unweighted UniFrac distances | There was no significant difference in gut microbial community composition between the groups. | *P*=0.078 |
|  | ACE | There was no significant difference between the groups. | *P*=0.08 |  |  |  |
|  | Richness | There was no significant difference between the groups. | *P*=0.07 |  |  |  |
|  | Phylogenetic diversity | There was no significant difference between the groups. | *P*=0.06 |  |  |  |
| McEachron 2022 | Shannon | The CP group had lower Shannon diversity than HC. | *P*<0.001 | PCoA based on Bray-Curtis metrics | There was a significant difference in gut microbial community composition between the groups. | *P*=0.002 |
| Frost 2020 | Shannon | The CP group had lower Shannon diversity than HC. | *P*<0.05 | PCoA based on Bray-Curtis metrics | There was a significant difference in gut microbial community composition between the groups. | *P*<0.001 |
|  | Simpson | The CP group had lower Simpson diversity than HC. | *P*<0.05 |  |  |  |
| Wang 2020 | Shannon | The CP group had lower Shannon diversity than HC. | *P*<0.01 | PCoA | There was a significant difference in gut microbial community composition between the groups. | *P*<0.05 |
|  | Simpson | The CP group had higher Simpson diversity than HC. | *P*<0.01 | NMDS | There was a significant difference in gut microbial community composition between the groups. | *P*<0.05 |
| Zhou 2020 | Observed species | The CP group had less observed species than HC. | *P*=0.001 | PCoA of weighted UniFrac distances | There was no significant difference in gut microbial community composition between the groups. | *P*=0.197 |
|  | Shannon | The CP group had lower Shannon diversity than HC. | *P*=0.001 |  |  |  |
|  | Simpson | The CP group had higher Simpson diversity than HC. | *P*=0.026 | PCoA of unweighted UniFrac distances | There was a significant difference in gut microbial community composition between the groups. | *P*=0.004 |
|  | ACE | The CP group had lower ACE than HC. | *P*=0.003 |  |  |  |
|  | Richness | The CP group had lower Richness than HC. | *P*=0.001 |  |  |  |
|  | Phylogenetic diversity | The CP group had lower Phylogenetic diversity than HC. | *P*=0.003 |  |  |  |
| Ciocan 2018 | Shannon | The CP group had lower Shannon diversity than HC. | *P*<0.05 | PCoA of weighted UniFrac distances | There was a significant difference in gut microbial community composition between the groups. | *P*=0.001 |
|  |  |  |  | PCoA of unweighted UniFrac distances | There was a significant difference in gut microbial community composition between the groups. | *P*=0.001 |
| Jandhyala 2017 | Richness | The CP group had lower Richness than HC. | *P*=0.01 | PCA | There was a significant difference in gut microbial community composition between the groups. | *P*=0.009 |
|  | Shannon | The CP group had lower Shannon diversity than HC. | *P*=0.04 |  |  |  |
| **3. CP vs. PDAC** | | | | | | |
| Chen 2023 | Richness | There was no significant difference between the groups. | *P*=0.19 | PCoA of weighted UniFrac distances | There was a significant difference in gut microbial community composition between the groups. | *P*=0.002 |
|  | Observed species | There was no significant difference between the groups. | *P*=0.24 |  |  |  |
|  | Phylogenetic diversity | There was no significant difference between the groups. | *P*=0.3 |  |  |  |
| Kartal 2022 | Richness | There was no significant difference between the groups. | *P*=0.19 | RDA based on Bray-Curtis distance | There was a mild but significant difference in gut microbial community composition between the groups. | *P*=0.003 |
|  | Shannon | There was no significant difference between the groups. | *P*=0.093 |  |  |  |
|  | Simpson | There was no significant difference between the groups. | *P*=0.15 |  |  |  |
|  | Eveness | There was no significant difference between the groups. | *P*=0.1 |  |  |  |
| Zhou 2021 | Richness | There was no significant difference between the groups. | *P*=0.082 | PCoA based on Bray-Curtis metrics | There was a significant difference in gut microbial community composition between the groups. | *P*=0.004 |
|  | Shannon | There was no significant difference between the groups. | *P*=0.057 |  |  |  |
|  | Simpson | There was no significant difference between the groups. | *P*=0.051 |  |  |  |
|  | Eveness | There was no significant difference between the groups. | *P*=0.12 |  |  |  |

PDAC, pancreatic ductal adenocarcinoma; HC, Healthy Controls; ND, Not Declared.

PCA, Principal Component Analysis; PCoA, Principal Coordinate Analysis; RDA, Redundancy Analysis.; MDS, Multidimensional Scaling; NMDS, Nonmetric Multidimensional Scaling; ACE, Abundance-based Coverage Estimator;
