# Supplementary material for: Gut microbiome changes associated with chronic pancreatitis and pancreatic cancer: a systematic review and meta-analysis
Source: Int J Surg. 2024 Jun 7;110(9):5781–94. doi: 10.1097/JS9.0000000000001724 (PMC11392207; doi:10.1097/JS9.0000000000001724)
Supplement: Supplementary file 6 [file js9-110-5781-s006.docx]

**Supplementary Fig. 1. Forest plot of meta-analysis of the alpha-diversity**

1. Forest plot of meta-analysis of the alpha-diversity (pancreatic cancer versus healthy individuals) (a) Shannon Index; (b) Simpson Index; (c) Evenness; (d) Richness.


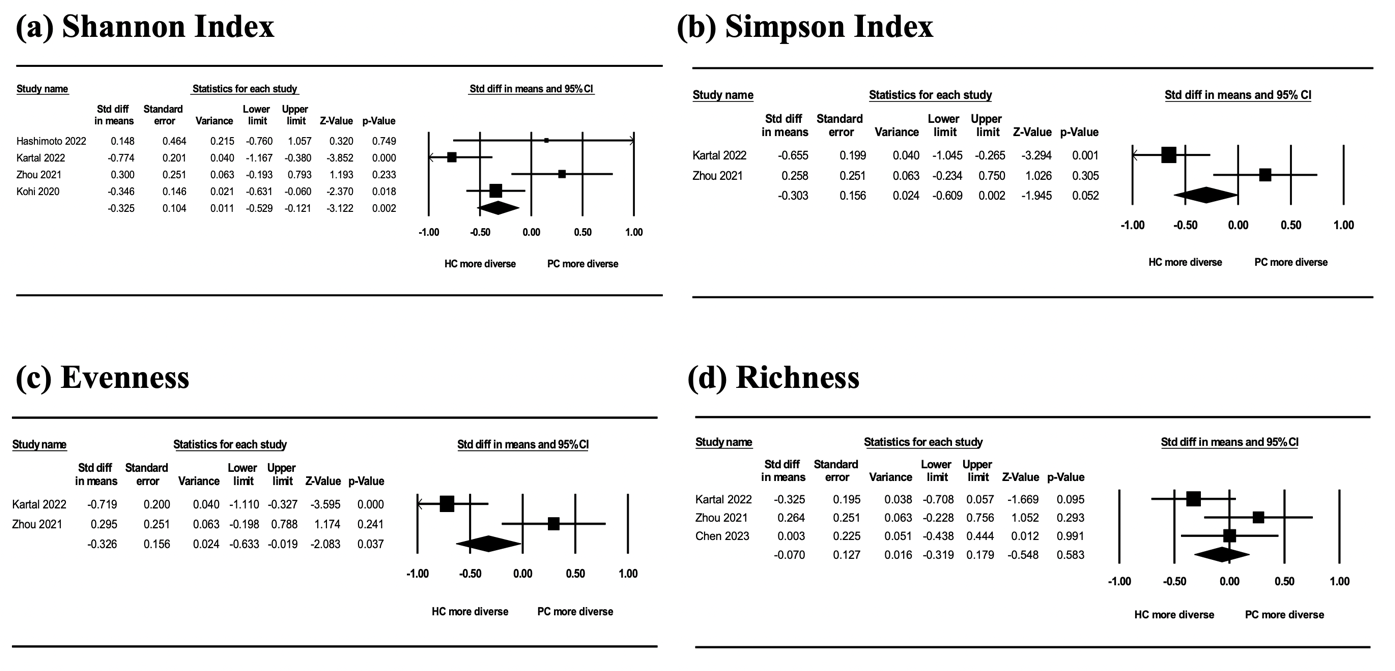


1.
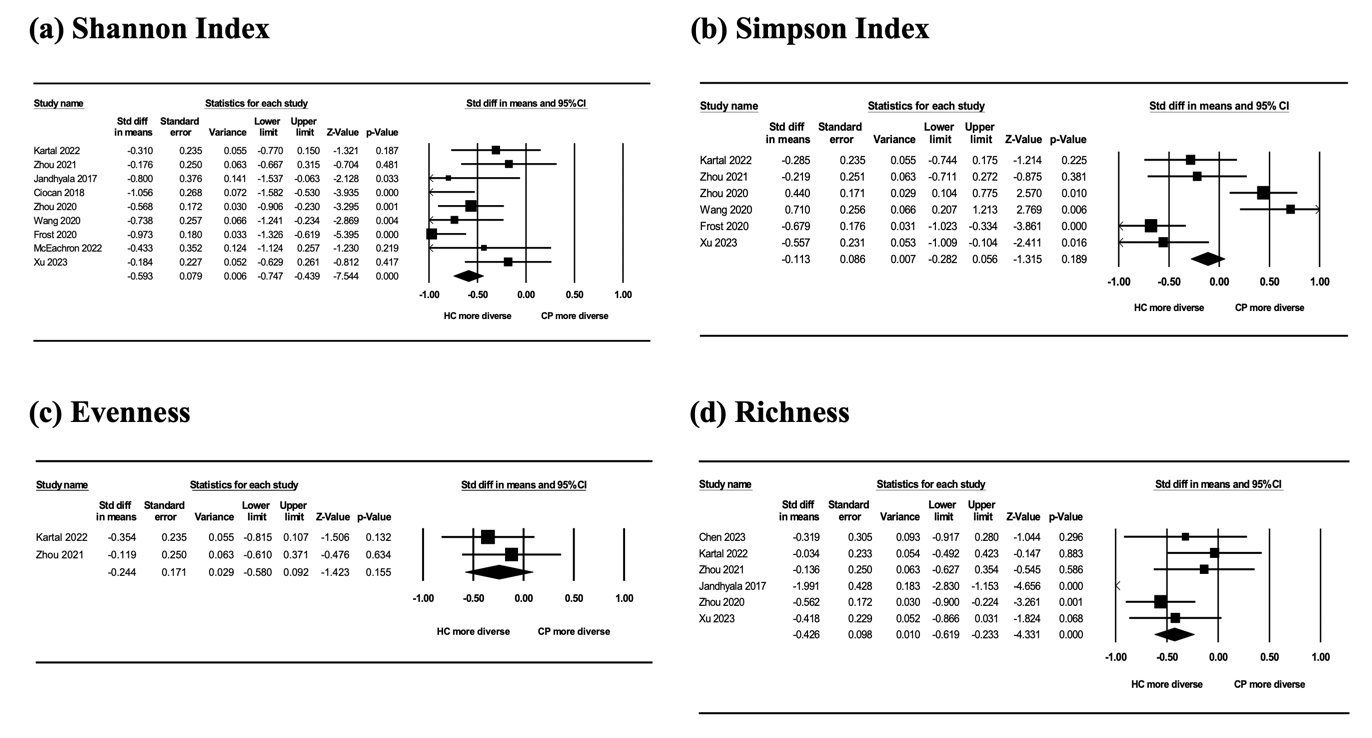
Forest plot of meta-analysis of the alpha-diversity (chronic pancreatitis versus healthy individuals) (a) Shannon Index; (b) Simpson Index; (c) Evenness; (d) Richness.
2.
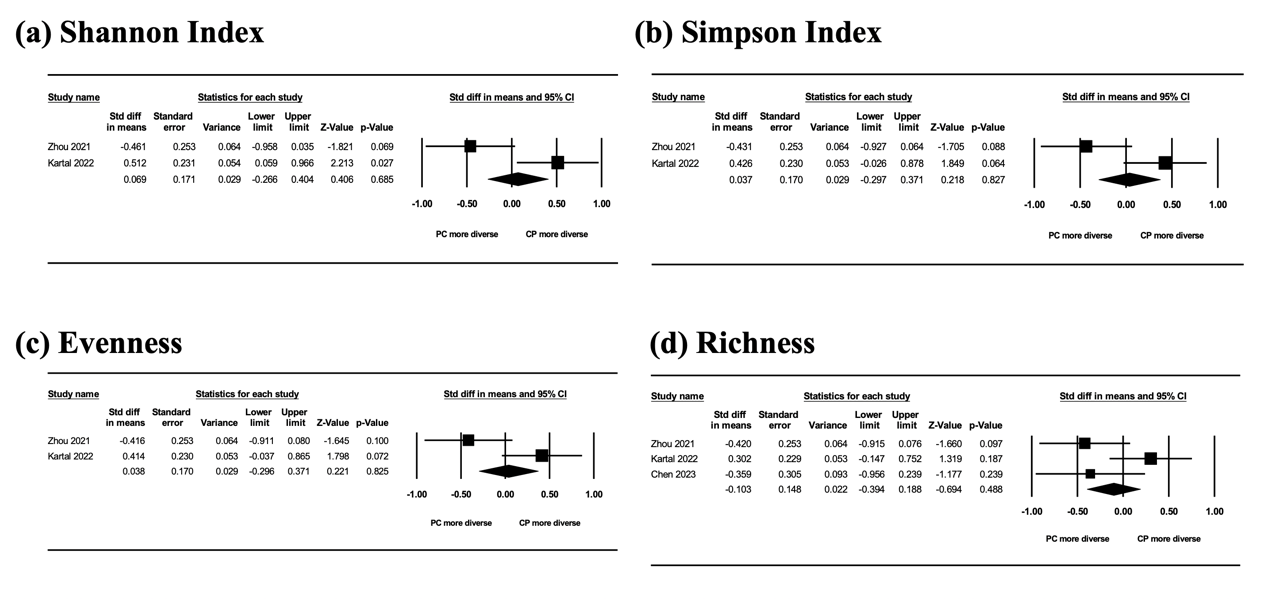
Forest plot of meta-analysis of the alpha-diversity (chronic pancreatitis versus pancreatic cancer) (a) Shannon Index; (b) Simpson Index; (c) Evenness; (d) Richness.
