# Supplementary material for: Gut microbiome changes associated with chronic pancreatitis and pancreatic cancer: a systematic review and meta-analysis
Source: Int J Surg. 2024 Jun 7;110(9):5781–94. doi: 10.1097/JS9.0000000000001724 (PMC11392207; doi:10.1097/JS9.0000000000001724)
Supplement: Supplementary file 7 [file js9-110-5781-s007.docx]

**Supplementary Table 6.** The heterogeneity (*I^2^*)of pooled results of alpha-diversity and gut microbiota composition.

| Comparison | Outcomes | *I^2^* value |
| --- | --- | --- |
| **PDAC vs. HC** | Shannon Index | 75.475 |
|  | Simpson Index | 88.112 |
|  | Evenness | 89.962 |
|  | Richness | 70.923 |
| **CP vs. HC** | Shannon Index | 49.104 |
|  | Simpson Index | 85.917 |
|  | Evenness | 0.000 |
|  | Richness | 72.674 |
| **CP vs. PDAC** | Shannon Index | 87.574 |
|  | Simpson Index | 84.070 |
|  | Evenness | 83.029 |
|  | Richness | 62.961 |
| **PDAC vs. HC** | Actinbacteria | 0.000 |
|  | Bacteroidetes | 0.000 |
|  | Firmicutes | 0.000 |
|  | Fusobacteria | 0.000 |
|  | Proteobacteria | 0.000 |
| **CP vs. HC** | Actinbacteria | 0.000 |
|  | Bacteroidetes | 0.000 |
|  | Firmicutes | 0.000 |
|  | Proteobacteria | 16.930 |
| **CP vs. PDAC** | Bacteroidetes | 0.000 |
|  | Firmicutes | 0.000 |
|  | Proteobacteria | 38.583 |

PDAC, Pancreatic Ductal Adenocarcinoma; PC, Pancreatic Cancer; CP, Chronic Pancreatitis; HC, Healthy Controls.
