# Supplementary material for: Gut microbiome changes associated with chronic pancreatitis and pancreatic cancer: a systematic review and meta-analysis
Source: Int J Surg. 2024 Jun 7;110(9):5781–94. doi: 10.1097/JS9.0000000000001724 (PMC11392207; doi:10.1097/JS9.0000000000001724)

**Supplementary Fig. 2.** **Sensitivity analysis of meta-analysis of the composition of gut microbiota at the phylum level.**


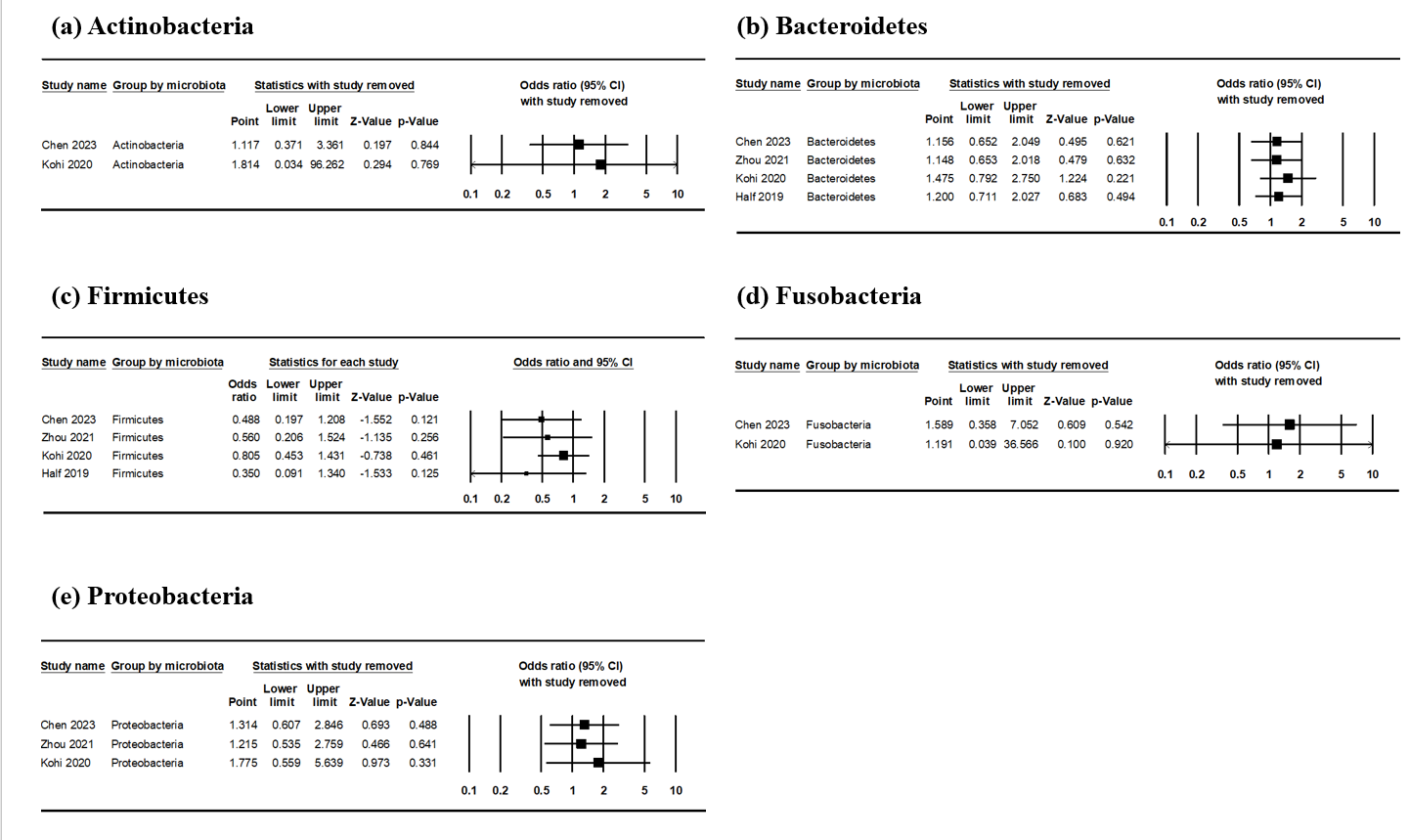
(A) Sensitivity analysis of meta-analysis of the composition of gut microbiota at the phylum level (pancreatic cancer versus healthy individuals). (a) Actinobacteria; (b) Bacteroidetes; (c) Firmicutes; (d) Fusobacteria; (d) Proteobacteria.


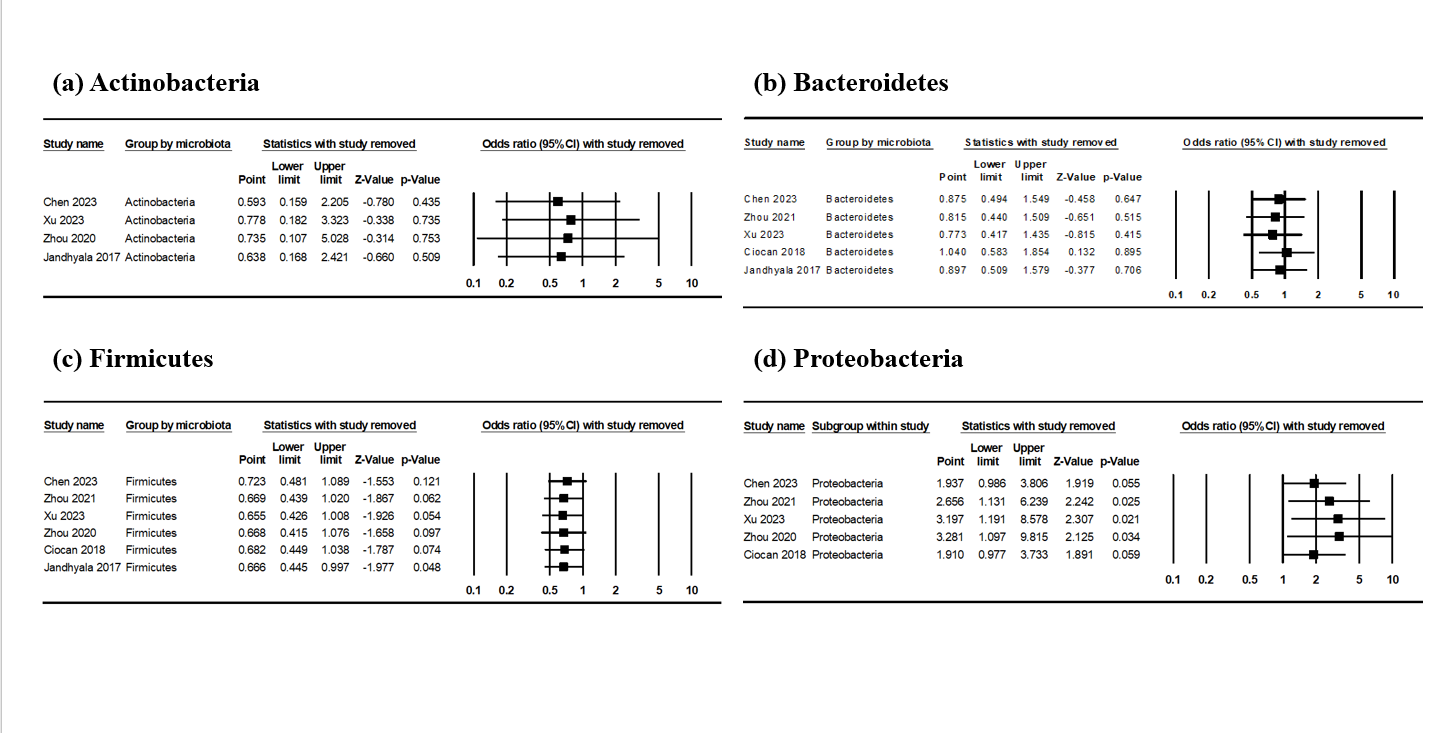
(B) Sensitivity analysis of meta-analysis of the composition of gut microbiota at the phylum level (chronic pancreatitis versus healthy individuals). (a) Actinobacteria; (b) Bacteroidetes; (c) Firmicutes; (d) Proteobacteria.

(C) Sensitivity analysis of meta-analysis of the composition of gut microbiota at the phylum level (chronic pancreatitis versus pancreatic cancer). (a) Bacteroidetes; (b) Firmicutes; (c) Proteobacteria.


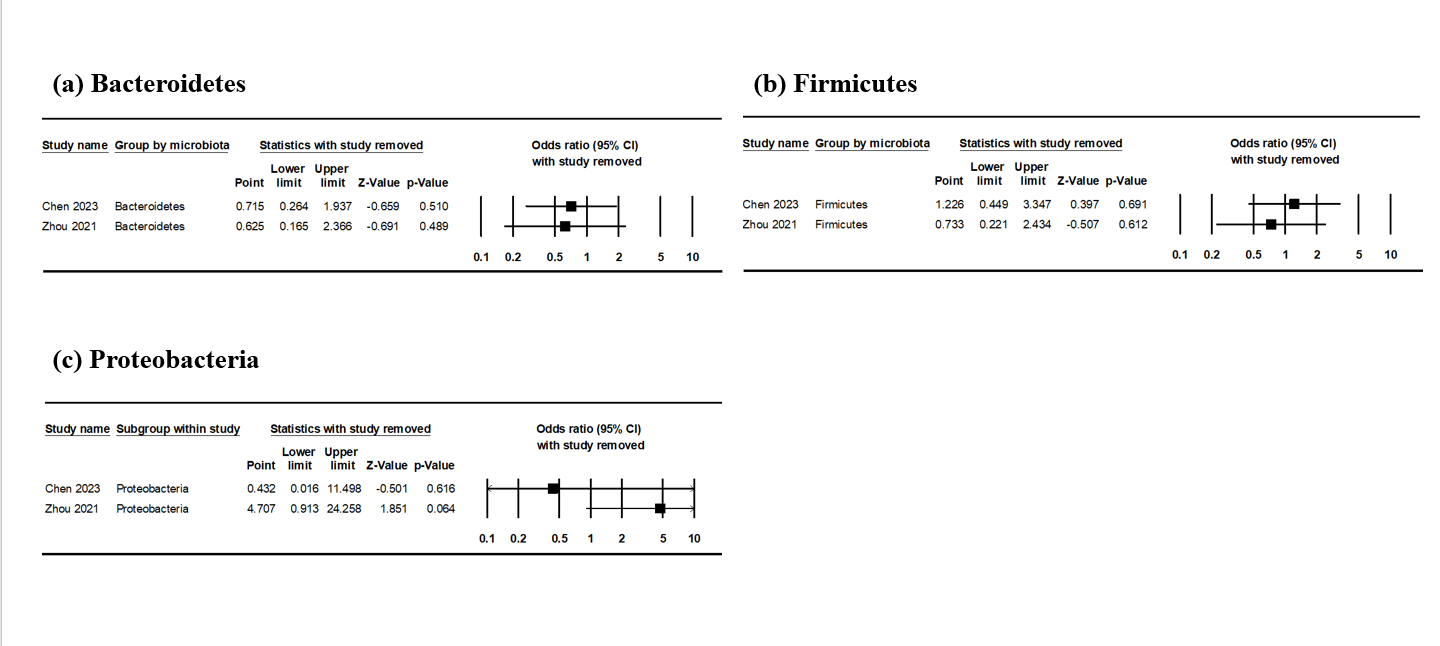

Supplement: Supplementary file 8 [file js9-110-5781-s008.docx]
